# Elevated serum levels of interleukin-17A in children with autism

**DOI:** 10.1186/1742-2094-9-158

**Published:** 2012-07-02

**Authors:** Laila Yousef AL-Ayadhi, Gehan Ahmed Mostafa

**Affiliations:** 1 Department of Physiology, Autism Research and Treatment Center, Al-Amodi Autism Research Chair, Faculty of Medicine, King Saud University, Riyadh, Saudi Arabia; 2 Department of Pediatrics, Faculty of Medicine, Ain Shams University, Cairo, Egypt; 3 9 Ahmed El-Samman Street off Makram Ebaid, Nasr City, Cairo, Egypt

**Keywords:** Autism, Autoimmunity, Childhood autism rating scale, IL-17A, T-helper 17

## Abstract

**Background:**

The T-helper (Th)1/Th2 dichotomy dominated the field of immune regulation until interleukin (IL)-17-expressing T cells (Th17) were proposed to be a third lineage of helper T cells, the key players in the pathogenesis of autoimmune disorders. Autoimmunity to brain tissue may play a pathogenic role in autism. IL-17A is a pro-inflammatory cytokine that has been shown to play an important role in various autoimmune neuroinflammatory diseases. The aim of this study was to measure serum levels of IL-17A in relation to the degree of the severity of autism.

**Methods:**

Serum IL-17A levels were measured by ELISA in 45 children with autism and 40 matched healthy controls.

**Results:**

Children with autism had significantly higher serum IL-17A levels than healthy controls (*P* <0.001), with increased serum levels of IL-17A found in 48.9% of the autism group. Patients with severe autism had significantly higher serum IL-17A levels than those with mild to moderate autism (*P* = 0.01), and raised serum IL-17A levels were significantly more common in children with severe autism (67.9%) than in those with mild to moderate autism (17.6%), *P* = 0.001.

**Conclusions:**

Serum IL-17A levels were raised in the group with autism, and the levels correlated significantly with the severity of autism. This is the first study to measure levels of IL-17A in relation to the severity of autism, to our knowledge. Further research, with a larger subject population, is warranted to determine whether the increase of serum IL-17A levels plasma has a pathogenic role in autism, and whether anti- IL-17A therapy could be useful

## Background

CD4-positive T lymphocytes play a major role in the regulation of adaptive immunity. Upon activation by antigen-presenting cells (APC), naive antigen-specific CD4+ T cells differentiate into different subsets of T cells. In addition to the classic T-helper (Th)1 and Th2 cells, several novel effector T cell subsets have been recently identified, including Th17 cells [1].

Th1 and Th2 cells are differentially induced, and regulate immunity against intracellular and extracellular pathogens, respectively. They are also involved in immune pathologies such as autoimmunity and allergy [2,3]. Th17 cells have been shown to induce host protection against extracellular pathogens and tissue inflammation [4-6], and are thought to play a key role in autoimmune diseases, including the tissue injury associated with these conditions [7,8].

Th17 CD4 T cells are characterized by production of the cytokines interleukin (IL)-17A (IL-17) and IL-17 F, and IL-21 and IL-22 [9]. Since their discovery in 2003, there have been numerous studies on Th17 cells, and they have emerged as key players in the pathogenesis of some autoimmune neuroinflammatory diseases and other autoimmune disorders traditionally attributed to Th1 cells. This may be attributable to the induction of neutrophil-recruiting chemokines (chemokine (C-X-C motif) ligand (CXCL)1, CXCL2, CXCL8) by IL-17 [10,11].

Autoimmunity may have a role in the pathogenesis of autism in a subgroup of patients. Brain-specific auto-antibodies have been found in some children with autism [12-18], and there is also an increase in the frequency of autoimmune disorders in autistic families [19-26]. Although the origins of autoimmunity in autism are unknown, it is thought that the major histocompatibility complex genes and their products might be involved [23,27,28].

In this study, we aimed to measure serum IL-17A levels in a group of children with autism, and to examine the relationship between serum levels of IL-17A and the degree of the severity of autism.

## Methods

### Ethics approval

The local ethics committee of the Faculty of Medicine, King Saud University, Riyadh, Saudi Arabia, approved the study, and informed written consent for participation in the study was signed by the parents or the legal guardians of the participants.

### Study population

This was a cross-sectional study, which recruited children with autistic disorder from the Autism Research and Treatment Center, King Khalid University Hospital (Riyadh, Saudi Arabia). Patients fulfilled the criteria of the diagnosis of autism based on the *Diagnostic and Statistical Manual of Mental Disorders*, fourth edition [29]. Patients with associated neurological diseases (such as cerebral palsy and tuberous sclerosis) and metabolic disorders (such as phenylketonuria) were excluded form the study.

In total, 45 children (36 male, 9 female; age (mean ± SD) = 8.44 ± 1.73 years, range 6 to 11 years) were enrolled in the study group.

The control group comprised 40 age- and sex-matched, apparently healthy children (32 male, 8 female; age (mean ± SD) = 8.33 ± 1.72 years, range 6 to 11). These children were the healthy older siblings of healthy infants who were attending the Well Baby Clinic at King Khalid University Hospital for routine follow-up of their growth parameters. The control children were not related to the children with autism, and had no clinical indications of infectious disease or neuropsychiatric disorders. They included none of the enrolled patients with autism and healthy control children had any clinical indications of autoimmune disorders such as arthralgia, arthritis, fever of unknown origin, shin rash, purpuric eruption, photosensitivity, excessive hair loss, hematuria or oliguria. All subjects had normal results for urine analysis and erythrocyte sedimentation rate.

### Study measurements

#### Clinical evaluation of autistic patients

Evaluation of the study group was based on clinical history-taking from caregivers, and on clinical examination, and neuropsychiatric assessment of the patients. In addition, the degree of the disease severity was assessed by the Childhood Autism Rating Scale (CARS) [30] which rates the child on a four-point scale in each of fifteen areas (relating to people; emotional response; imitation; body use; object use; listening response; fear or nervousness; verbal communication; non-verbal communication; activity level; level and consistency of intellectual response; adaptation to change; visual response; taste, smell and touch response; and general impressions). A score of 30 to 36 points on this scale indicates mild to moderate autism (n = 17), and a score of 37 to 60 points indicates severe autism (n = 28).

#### Assessment of serum interleukin-17A levels

Serum levels of IL-17A were evaluated using an ELISA kit (R & D Systems, Inc. 614 McKinley Place NE, Minneapolis, MN 55413, USA) designed to measure human IL-17A in serum. Blood samples were collected from each studied subject; 2 mL of blood were placed into a dry clean tube and left to clot at room temperature, then separated by centrifugation for 15 minutes. The serum was removed and stored at −20°C until required. Repeated freeze–thaw cycles were avoided to prevent loss of bioactive substances.

Microtiter strips coated with a monoclonal antibody specific for IL-17A were used. Samples, including standards of known IL-17A concentrations were placed in the wells of these strips. Two hundred uL of IL-17A antigen were added to the wells, and the strips were incubated for 30 minutes at room temperature. Strips were washed for 3 times in 400 ul of wash buffer then a biotinylated monoclonal antibody specific for IL-17A was added and the plate was incubated for one hour at room temperature. Streptavidin peroxidase was then added. After incubation and washing to remove all unbound enzyme, a substrate solution (color reagents A and B mixed together in equal volumes within 15 minutes of use) that acts on the bound enzyme was added to induce a colored reaction product, the intensity of which is directly proportional to the concentration of IL-17A present in the sample. To increase accuracy, all samples were analyzed twice in two independent experiments to assess interassay variations and to ensure reproducibility of the results (*P* >0.05). No significant crossreactivity or interference was seen.

### Statistical analysis

The results were analyzed by commercially available software package (Statview, Abacus Concepts Inc., Berkley, CA, USA). The data were non-parametric, thus they are presented as median and interquartile range (IQR; 25th to 75th percentile). Patients were considered to have raised serum IL-17A if the levels were above the highest cut-off values (1.73 pg/ml), which was the 95th percentile of the serum IL-17A levels of healthy controls, as the distribution of the data was non-parametric. The Mann–Whitney test was used for comparison between these data, and the χ² test was used for comparison between qualitative variables of the studied groups. Spearman’s rho correlation coefficient ‘r’ was used to determine the relationship between different variables. For all tests, *P* <0.05 was considered significant.

## Results

**Figure 1:**
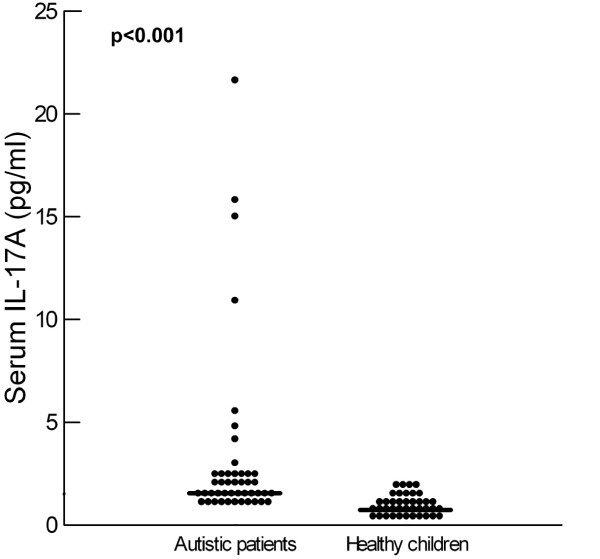
**Serum levels of IL-17A in autistic patients and healthy children.** Median value for each group is shown by a horizontal bar.

Children with autism had significantly higher serum IL-17A levels than healthy controls (*P* <0.001(Table 1, Figure 1). Increased serum IL-17A levels were found in 48.9% (22/45) of the patients with autism.

**Table 1:** Serum levels of IL-17A in autistic children and their relation to the severity of autism

	**Serum IL-17A (pg/ml),**	**Z score**
	**median (IQR)**	**P value**
Healthy children (n = 40)	0.77 (0.8)	6.4
Patients with autism (n = 45)	1.7 (0.9)	(< 0.001)
Patients with mild to moderate autism (n = 17)	1.5 (0.4)	2.52
Patients with severe autism (n = 28)	2.05 (2.3)	(0.01)

IL, interleukin.

**Figure 2:**
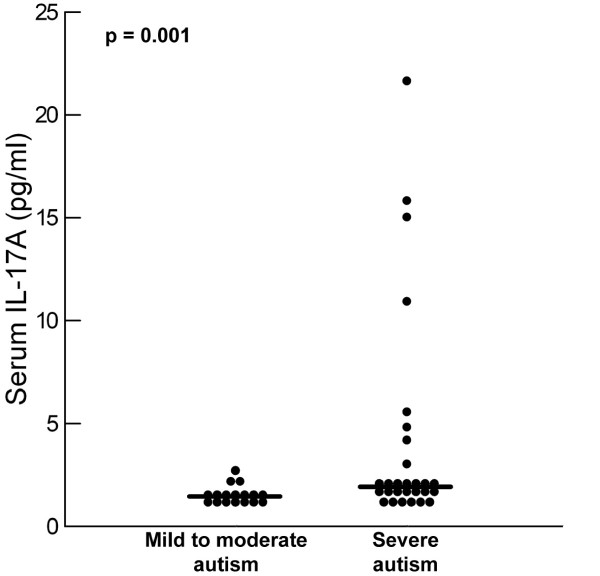
**Serum levels of IL-17A in relation to the degree of the severity of autism.** Median value for each group is shown by a horizontal bar.

Patients with severe autism had significantly higher serum IL-17A levels than children with mild to moderate autism (*P* = 0.01) (Table 1, Figure 2), and the frequency of increased serum IL-17A levels was significantly higher in children with severe autism (19/28; 67.9%) than in patients with mild to moderate autism (3/17; 17.6%) (*P* = 0.001).

There was no difference in serum IL-17A with regard to gender (median (IQR) = 1.82 (0.9) pg/ml for males and 1.7 (5.2) ng/ml for females; *P* = 0.32) or age of the children with autism (*P* = 0.32).

## Discussion

Th17 cells have been implicated in the pathogenesis of autoimmune disease, findings supported by recent clinical trials using anti-IL-17 in the treatment of these diseases [31]. Th17 cells are characterized by their production of IL-17 cytokines, which control the inflammatory responses by triggering the secretion of pro-inflammatory cytokines and chemokines [5,32].

In our series, children with autism had significantly higher serum IL-17A levels than healthy controls (*P* <0.001). Raised serum IL-17A levels were found in 48.9% (22/45) of the patients with autism. A recent study reported significantly higher plasma concentrations of IL-17 in 28 male subjects with high-functioning autism spectrum disorder (ASD) compared with 28 matched controls, and the patients with ASD also had higher plasma concentrations of other cytokines such as IL-1 receptor antagonist, IL-1β, IL-5, IL-8, IL-12 and IL-13 compared with controls. The authors suggested that abnormal immune responses, as assessed by multiplex analysis of cytokines, might be a biological trait marker for ASD [33]. Another study investigated the cellular release of IL-17 and IL-23 after *in vitro* immunological challenge of peripheral blood mononuclear cells from children with ASD compared with age-matched, typically developing, controls, and found that the concentration of IL-23, but not IL-17, was significantly reduced in ASD compared with controls. Those authors recommended that decreased cellular IL-23 production in ASD warrants further research to determine its role in the generation and survival of Th17 cells, a cell subset important in neuroinflammatory conditions that may include ASD [34].

IL-17A is a pro-inflammatory cytokine that is produced by a number of human immune cells, including Th17 cells, neutrophils, and peripheral blood mononuclear cells. Studies have shown that IL-17A is upregulated and involved in the pathogenesis of various autoimmune inflammatory diseases such as rheumatoid arthritis, systemic lupus erythematosus, and systemic sclerosis [35-38]. In addition, IL-17A has been shown to play an important role in various autoimmune neuroinflammatory diseases such as multiple sclerosis (MS) and experimental autoimmune encephalomyelitis (EAE) [39].

In immune responses against infection and models of autoimmune diseases, Th1 and Th17 cells often develop simultaneously [3].The differentiation of Th1 or Th17 cells occurs after exposure to APC-derived polarizing cytokines such as IL-12 for Th1 cells [40] and transforming growth factor-β, IL-1β, IL-6 and IL-23 for Th17 cells [5,41-43]. These polarizing cytokines further induce the expression of the transcription factors T-bet or retinoic acid receptor-related orphan receptor (ROR)γt and RORα for Th1 and Th17 differentiation respectively [44,45]. Dendritic cells (DCs), which are activated by signals from pathogen-associated recognition receptors, recognize components of intracellular pathogens [46], induce the production of polarizing cytokines, and drive generation of Th17 responses. Consistent with these findings, Th17 cells have a role in immunity against intracellular pathogens. In addition, a clear role for IL-17 in generation of chemokine responses, induction of anti-microbial proteins and recruitment of neutrophils for control of extracellular pathogens has emerged [47]. The pro-inflammatory actions of Th17 cells ,both in the clearance of various pathogens and in autoimmunity, may occur through the induction of neutrophil-recruiting chemokines (CXCL1, CXCL2, CXCL8) by IL-17 [10,11].

This study is the first to measure serum IL-17 in relation to the disease severity in children with autistic disorder, to our knowledge. A few previous studies measured serum IL-17 in children with ASD, but these studies did not correlate serum IL-17 levels with the degree of severity of autism. In the present study, we found that patients with severe autism had significantly higher serum IL-17A levels than children with mild to moderate autism, *P* = 0.01I, and children with severe autism were also significantly more likely to have increased serum IL-17A (67.9%) than patients with mild to moderate autism (17.6%) (*P* = 0.001). This may indicate that the extent of the increase in serum IL-17A levels is closely linked to the degree of the severity of autism. However, it is not clear whether the increase in serum IL-17A levels is a mere consequence of autism or has a pathogenic role in the disease.

CD4+ CD25^high^ regulatory T-cells (Tregs) play an important role in the establishment of immunological self tolerance and thereby, in the prevention of autoimmunity [48]. Tregs can suppress Th17 cells and autoimmunity, as activation of Th17 cells can start tissue inflammation [49]. A recent study reported deficiency of Tregs in 73.3% of children with autism [25], an dthus, Deficiency of Tregs may be one of the reasons behind the activation of Th17 cells and the elevation of serum IL-17 A levels in children with autism. Osteopontin is a key pro-inflammatory mediator that may serve to perpetuate and amplify the inflammatory process in many autoimmune neuroinflammatory disorders such as EAE in mice and MS in humans [50]. The Th17-related response in humans is enhanced by osteopontin, which may contribute to the pro-inflammatory actions of Th17 cells in the clearance of pathogens and in autoimmunity [10,11]. A recent study reported increased serum osteopontin levels in 80.95% of children with autism [51]. Thus, increased serum osteopontin levels in autism may be another contributing factor to the elevation of serum IL-17A levels in children with autism.

Autoimmunity to brain tissue may have a pathogenic role in autism [52-55], as indicated by the presence of brain-specific auto-antibodies in some children with autism [12-18]. Levels of IL-12 and interferon (IFN)-γ have been reported to be significantly increased in patients with autism [56]. It was suggested that increased IL-12 and IFNγ may indicate stimulation of Th1 cells pathogenetically linked to autoimmunity in autism [56]. Recently, increased pro-inflammatory Th1 cytokines were reported to be associated with greater impairments in core features of autism [57]. Macrophages and DCs express IL-17A receptors, and can respond to IL-17, producing cytokines and chemokines [58,59]. These data suggest that IL-17 responses can regulate the induction and generation of Th1 responses. The mechanism by which IL-17 regulates the Th1 pathway seems to be via induction of IL-12 and IFN-γ in APCs. After IL-17 stimulation, DCs and macrophages produce IL-12 and IFN-γ, respectively. IL-17-dependent-DC-derived IL-12 was able to drive the differentiation of naive T cells into Th1 cells, and IL-17-dependent macrophage-derived IFN-γ was able to activate macrophages for control of intracellular pathogens [59]. Because IL-17A is a stimulator of pro-inflammatory cytokines, IFN-γ and IL-12 [59], it could be the inducer of the Th1 response that has been reported previously to be linked to autoimmunity in autism [56]. In addition, the possible role of IL-17A in induction of the production of brain auto-antibodies may be associated with induction of the production of neutrophil-recruiting chemokines [10,11]. Moreover, osteopontin, which was recently reported to be increased in many children with autism [51], induces myelin antigen-specific IL-17 production from Th17 cells via specific osteopontin receptors (β3 integrin receptors) on T cells [50].

IL-17A was reported to have a possible role in some autoimmune neuroinflammatory diseases [39], thus the increased serum levels of IL-17A found in our study may be a possible contributing factor to the increased frequency of the brain-specific auto-antibodies in some children with autism. Further research is warranted to determine the possible link between increased serum levels of IL-17A and the presence of brain-specific auto-antibodies in some children with autism.

## Conclusions

In this study, we found that serum IL-17A levels were increased in some children with autism, and were significantly correlated with the severity of autism. However, further research, with a larger subject population, is warranted to determine whether this increase in serum IL-17A levels plasma has a pathogenic role in autism. The role of anti- IL-17A therapy in autism should also be studied.

## Abbreviations

APC, Antigen-presenting cells; CARS, Childhood Autism Rating Scale; CXCL, Chemokine (C-X-C motif) ligand; DCs, Dendritic cells; EAE, Experimental autoimmune encephalomyelitis; ELISA, enzyme-linked immunosorbent assay; IFN-γ, Interferon-gamma; IL, Interleukin; IQR, Interquartile range; MS, Multiple sclerosis; ROR, retinoic acid receptor-related orphan receptor; Th, T-helper; Tregs, CD4+ CD25high regulatory T-cells.

## Competing interests

The authors declare that they have no competing interests.

## Authors’ contributions

Both authors designed and performed the research and wrote the manuscript, and both have read and approved the final manuscript.
